# An exploratory cohort study of sensory extinction in acute stroke: prevalence, risk factors, and time course

**DOI:** 10.1007/s00702-016-1663-x

**Published:** 2016-12-09

**Authors:** Joseph Kamtchum-Tatuene, Gilles Allali, Arnaud Saj, Thérèse Bernati, Roman Sztajzel, Pierre Pollak, Isabelle Momjian-Mayor, Andreas Kleinschmidt

**Affiliations:** 1 Neurology Division, Department of Clinical Neurosciences, Geneva University Hospital, Geneva, Switzerland; 2 Brain Infections Group, Institute of Infection and Global Health, University of Liverpool, 8 West Derby Street, Liverpool, L69 7BE UK; 3 Division of Cognitive and Motor Aging, Department of Neurology, Albert Einstein College of Medicine, Yeshiva University, Bronx, NY USA; 4 Neurology and Cognitive Imaging Laboratory, Faculty of Medicine, University of Geneva, Geneva, Switzerland; 5 Neurology and Neurophysiology Centre, Geneva, Switzerland

**Keywords:** Acute stroke, Sensory extinction, Visuospatial neglect, Risk factors, Prognosis

## Abstract

Most studies on sensory extinction have focused on selected patients with subacute and chronic right hemisphere lesions. In studies conducted on acute stroke patients, risk factors and time course were not evaluated. Our aim was to determine the prevalence, risk factors, and time course of sensory extinction in the acute stroke setting. Consecutive patients with acute stroke were tested for tactile, visual, auditory, and auditory-tactile cross-modal extinction, as well as for peripersonal visuospatial neglect (PVN). Tests were repeated at 2, 7, 15, 30, and 90 days after initial examination. A multivariable logistic regression analysis was performed to test the association between sensory extinction and demographic and clinical risk factors. Seventy-three patients (38.4% women) were recruited: 64 with ischemic stroke and nine with haemorrhagic stroke. Mean age was 62.3 years (95% CI 58.8–65.7), mean NIHSS score was 1.6 (95% CI 1.2–2.1), and mean time to first examination was 4.1 days (95% CI 3.5–4.8). The overall prevalence of all subtypes of sensory extinction was 13.7% (95% CI 6.8–23.8). Tactile extinction was the most frequent subtype with a prevalence of 8.2% (95% CI 3.1–17.0). No extinction was found beyond 15 days after the first examination. After adjustment for age, sex, lesion side, type of stroke, time to first examination and stroke severity, a lesion volume ≥2 mL (adjusted OR = 38.88, *p* = 0.04), and presence of PVN (adjusted OR = 24.27, *p* = 0.04) were independent predictors of sensory extinction. The insula, the putamen, and the pallidum were the brain regions most frequently involved in patients with sensory extinction. Extinction is a rare and transient phenomenon in patients with minor stroke. The presence of PVN and lesion volume ≥2 mL are independent predictors of sensory extinction in acute stroke.

**Electronic supplementary material:**

The online version of this article (doi:10.1007/s00702-016-1663-x) contains supplementary material, which is available to authorized users.

## Introduction

Extinction is a behavioural symptom of brain lesions where patients report, respond, or orient to a stimulus presented on the contralesional side, but fail to detect the same stimulus when presented concurrently with another stimulus on the ipsilesional side (Bender 1952; Chechlacz et al. 2014; de Haan et al. 2012). Extinction frequently co-occurs with visuospatial neglect and the question of whether they should be considered as common or separate deficits is still debated. Indeed, several authors still consider extinction as a milder form or a residual manifestation of neglect after recovery (Heilman and Valenstein 2012; Liu et al. 2011; Vuilleumier and Rafal 2000), while others support the idea that extinction and neglect are separate deficits (Karnath and Rorden 2012; Priftis et al. 2013; Vossel et al. 2011). The latter view relies on the fact that both syndromes can occur independently (Cocchini et al. 1999; Di Pellegrino and De Renzi 1995) and have frequently been related to distinct neuroanatomical substrates (Karnath et al. 2003; Karnath and Rorden 2012; Vallar et al. 1994).

Neglect can be sensory, visuospatial, motor, representational, or personal (Heilman et al. 2012; Punt et al. 2013; Saj and Vuilleumier 2013), and extinction can be motor or sensory. Sensory extinction can be further classified as unimodal (tactile, visual, or auditory), multimodal, or cross-modal (Jacobs et al. 2011). Unbalanced attentional competition between brain hemispheres (Driver and Vuilleumier 2001; Kinsbourne 1977; Riddoch et al. 2009) and impaired processing of contralesional sensory stimuli in the absence of a primary sensory deficit (Chechlacz et al. 2014; Rorden et al. 2009; Watling et al. 2013) are the mechanisms most frequently proposed to explain the occurrence of extinction. Such putative mechanisms can explain the classical extinction scenario where contralesional stimuli are extinguished in the context of double simultaneous stimulation, but recent descriptions of “anti-extinction” (Humphreys et al. 2002; Watling et al. 2013) and “ipsilesional extinction” (de Haan et al. 2015) suggest more complex pathomechanisms also involving a non-spatial deficit of attentional capacity at least in some patients with extinction. Anti-extinction occurs when there is poor report of a single stimulus presented on the contralesional side of space, but better report of the same item when it occurs concurrently with a stimulus on the ipsilesional side (Humphreys et al. 2002). Ipsilesional sensory extinction refers to failure to report an ipsilesional stimulus when presented simultaneously with a contralesional stimulus, while there is normal reporting of single ipsi- and contralesional stimuli (de Haan et al. 2015; Karnath 1988). Moreover, recent demonstrations of sensory extinction in healthy individuals highlight the existence of multisensory integration neuronal networks whose impairment could lead to altered perception of stimuli from either side of space no matter on which side a brain lesion is located (Jacobs et al. 2011). Lesions of multimodal neurons could be involved in the pathophysiology of ipsilesional cross-modal extinction by inducing mislocalization or misidentification of stimuli (Liu et al. 2011).

Most studies on extinction have been conducted on selected patients with subacute and chronic brain lesions (Chechlacz et al. 2014; Vallar et al. 1994; Vuilleumier 2013). The rare studies conducted on patients with acute stroke did not evaluate risk factors and time course of extinction (Becker and Karnath 2007) and some only reported patients with right hemisphere lesions (Umarova et al. 2011; Vallar et al. 1994; Vossel et al. 2011). Our aim here was, therefore, to gather data on prevalence, risk factors, and time course of sensory extinction in the acute stroke setting. Such data might help to improve our understanding of behavioural manifestations in acute stroke. They could also help generate new hypotheses for further studies on the pathophysiology of sensory extinction.

## Materials and methods

### Study design, setting, and selection of participants

This prospective cohort study was conducted on consecutive patients with acute stroke admitted to the Stroke Unit of Geneva University Hospital, from September 2012 to March 2014. The study procedure has been described previously (Kamtchum Tatuene et al. 2016). Briefly, exclusion criteria were past history of stroke, severe aphasia, and severe stroke (NIHSS score >20); and any documented alteration of visual, tactile, auditory, or cognitive functions susceptible to interfere with the neuropsychological evaluation. For instance, patients with clearly defined hemianopia, hemianesthesia, or hemihypoesthesia were not included in this study. Patients with altered level of consciousness were not systematically excluded but rather examined later depending on their capacity to cooperate. Patients were clinically tested for sensory extinction as soon as possible after their admission (visit 1). Five subtypes of sensory extinction were considered: homologous and heterologous tactile extinction as well as visual, auditory, and auditory-tactile cross-modal extinction. To perform the clinical assessment of patients, one of us (JKT) trained and evaluated three examiners.

During visit 1, we also recorded data on factors potentially related to sensory extinction: age, gender, handedness, stroke severity on the examination day as assessed by the National Institute of Health Stroke Scale (NIHSS) (Brott et al. 1989), presence of peripersonal visuospatial neglect (PVN), and stroke type (ischemic/hemorrhagic). We also determined lesion volume in mL (provided by the MRIcro software, see “Anatomical study and mapping of brain lesions”) and location (right/left hemisphere or bilateral) on plain computed tomography (CT) scans (for haemorrhagic stroke) or Diffusion-Weighted-Imaging sequences (DWI) obtained with a 3 T Magnetic Resonance Imaging (MRI) scanner within 15 days of symptoms onset (for ischaemic stroke).

All patients diagnosed with sensory extinction on visit 1 were then systematically re-evaluated 2 (visit 2), 7 (visit 3), 15 (visit 4), 30 (visit 5), and 90 (visit 6) days later. Follow-up was terminated once a patient did not present any type of sensory extinction on two consecutive visits.

### Procedures for the neuropsychological evaluation

During clinical assessment, patients were seated either in bed or, when possible, at a desk. They were requested to keep their eyes closed except when tested for visual extinction or when performing paper-and-pencil tasks. For each unimodal sensory extinction task (tactile, auditory or visual), stimulation sequences were established in advance and comprised ten unilateral (five ipsilesional and five contralesional) and ten bilateral simultaneous stimuli. These stimuli were randomly distributed in time (fixed random schedule) with the exception that the first one was always ipsilesional (following the common medical practice of assessing the unaffected side first). The number of correct answers for each type of stimulation was recorded. Regarding cut-off values, we could not rely on established rules from the literature as different decision thresholds have been used (Becker and Karnath 2007; Umarova et al. 2011; Vallar et al. 1994; Vossel et al. 2011). In this study, patients were classified as showing extinction if they met all the following three criteria: 100% correct answers for single ipsilesional stimuli, at least 80% correct answers for single contralesional stimuli and less than 80% correct answers for bilateral stimuli (Vallar et al. 1994). Patients showing less than 80% correct answers for single contralesional stimuli were considered as having a sensory deficit and were excluded.

Being aware that test results could be influenced by fluctuations of the strength of stimuli or asynchrony of stimulus onset and termination during bilateral stimulations, all neuropsychological tests were performed twice during each visit using non-standardized stimuli the first time and standardized stimuli the second time as described below.

#### Testing for homologous and heterologous tactile extinction

For these tasks, whenever possible, the examiner stood in front of the patient in the midsagittal plane or as close as possible to this plane for patients examined in bed. The tactile stimulus consisted of a brief slight touch applied by the examiner’s fingertip. When testing for homologous tactile extinction (same body part stimulated on both sides), the tactile stimulus was administered to the patient’s right or left cheek, or to both cheeks simultaneously. Before examination, patients were informed that stimuli could be single or double and that they were to give a verbal response (“single—right”, “single—left”, or “double”). When testing for heterologous tactile extinction, the stimulus was administered either to the patient’s ipsilesional cheek, the dorsal surface of the patient’s contralesional hand or the ipsilesional cheek, and the contralesional hand (dorsal surface), simultaneously. Before examination, patients were informed that stimuli could be single or double and that they were to give a verbal response (“single—cheek”, “single—hand”, or “double”).

#### Testing for visual extinction

We used the confrontation technique (Chechlacz et al. 2014; Umarova et al. 2011). The patient was instructed to keep looking at the examiner’s nose located at 60 cm distance in the midsagittal plane. The visual stimuli consisted of a brief movement (rapid flexion–extension) of the examiner’s index finger either in the right or left visual hemifield or simultaneously in both visual hemifields. The examiner’s right and left fingers were placed halfway between the examiner and the patient facing him, at 45° eccentricity on the horizontal plane. Before examination, patients were informed that stimuli could be single or double and that they were to give a verbal response (“single—right”, “single—left”, or “double”).

#### Testing for auditory extinction

The auditory stimuli consisted of a brief sound produced by the examiner, within 5 cm from the external auditory meatus, either on the right or left side or on both sides, simultaneously. This close distance was chosen, because extinction and other multimodal integration phenomena are known to be stronger in the near peripersonal space (Graziano et al. 1999; Jacobs et al. 2011). The sound was produced by snapping fingers. Before examination, patients were informed that stimuli could be single or double and that they were to give a verbal response (“sound—right”, “sound—left”, or “double”).

#### Testing for auditory-tactile cross-modal extinction

We used two sets of 20 stimulations. Each set was made up of ten unimodal stimulations (auditory or tactile as defined above) randomly administered either unilaterally or bilaterally, and ten cross-modal stimulations combining an auditory stimulus in one ear and a simultaneous tactile stimulus on the contralateral cheek. We decided to administer the tactile stimulus to the cheek rather than the hand, because the previous studies on visuo-tactile extinction suggested that cross-modal extinction is stronger when closely related body parts are stimulated (Farne et al. 2005; Jacobs et al. 2011). Unimodal stimulations considered as control were randomly intermixed with cross-modal stimulations. In case of cross-modal stimulation, the auditory stimulus was administered to the ipsilesional side for the first set of 20 stimulations and to the contralesional side for the second.

Before examination, patients were informed that stimuli could be single unimodal or double unimodal or double cross-modal. They had to give a verbal response (“touch—right”, “touch—left”, “touch - double”, “sound—right”, “sound—left”, “sound—double”, or “touch—sound”). The number of correct answers for each type of stimulation was recorded. Patients were classified as showing contralesional auditory-tactile cross-modal extinction if they gave less than 80% correct answers for cross-modal stimulations in the first set of 20 stimulations. They were classified as showing ipsilesional auditory-tactile cross-modal extinction if they gave less than 80% correct answers for cross-modal stimulations in the second set.

We used white noise rather than pure tones to maximise our chances of identifying cases of auditory-tactile cross-modal extinction. Previous studies have demonstrated that pure tones do not activate multimodal neurons and produce milder cross-modal extinction than white noise (Graziano et al. 1999; Ladavas et al. 2001). We decided to study auditory-tactile cross-modal extinction rather than visuo-tactile or audio-visual cross-modal extinctions, because auditory-tactile stimuli are easier to use in patients with acute stroke who usually feel tired and anxious. Had we chosen to study visuo-tactile extinction, we would have experienced difficulties related to positioning of patients and fatigue due to sustained fixation. Moreover, we thought that results found with auditory-tactile cross-modal extinction could be easily repeated when using other types of cross-modal extinction (Ladavas et al. 1998, 2001). Indeed, pathological findings could be aggravated due to the dominance of visual stimuli first described by Colavita in 1974 (Spence 2009).

#### Testing for peripersonal visuospatial neglect (PVN)

Two paper-and-pencil tasks were used: the Ota’s gap detection task (Ota et al. 2001) and a line bisection task (Azouvi et al. 2006). These tests were administered and interpreted as reported previously (Kamtchum Tatuene et al. 2016).

#### Standardization of testing procedures

Tactile, auditory, and auditory-tactile cross-modal extinction tasks were performed twice for each patient during each visit. Non-standardized tactile (slight touch with the tip of the index) and auditory (fingers snapping) stimuli were used the first time, whereas standardized tactile and auditory stimuli were used the second time. Standardized tactile stimuli were administered with a calibrated 5.07/10 g Semmes–Weinstein monofilament (Feng et al. 2009), while standardized auditory stimuli consisted of a preregistered click-like white noise administered through a headset connected to a computer. Examiners were trained for simultaneous administration of standardized stimuli during bilateral bimodal stimulations. The test for visual extinction was not standardized in this study.

### Anatomical study and mapping of brain lesions

The description of the location of brain lesions was done using region-involvement indices as reported previously (Kamtchum Tatuene et al. 2016). The following functional regions of the brain were attributed a score of 1 if they were partially or totally affected by the acute stroke or 0 if not involved at all: frontal, insular, rolandic, parietal, temporal, occipital, thalamic, caudate nucleus, putamen, pallidum, internal capsule, brain stem, and cerebellum. The region-specific score (RSS) was defined as the total number of times that a functional region had received a score of 1 after reviewing all the CT and MRI scans of patients with at least one subtype of sensory extinction. The region-involvement index (RII) was defined as the ratio of a RSS and the sum of all RSSs.

A lesion-overlap study was also performed to identify brain regions commonly damaged in patients with sensory extinction (Rousseaux et al. 2013; Saj et al. 2012; Verdon et al. 2010). Lesions identified on plain CT or DWI were manually reconstructed on a standardized brain template using the MRIcro software (http://www.mricro.com) (Karnath et al. 2011) to obtain a three-dimensional region of interest (ROI). The slices thickness was 2 mm. The ROI was then used to build an overlap map. The volume of each ROI (in cubic centimetres or millilitres) was automatically displayed in the bottom left corner of the ROI editing panel. All the analyses with MRIcro were done by a trained neuropsychologist who was blind of patients’ performance (AS).

### Ethical issues

The study was approved by the Geneva University Hospital Ethical Committee for Research on Human Beings (Authorization number: CER 12-191). All patients included gave written informed consent to take part in the study. Access to patients’ data and anonymized case report forms was restricted to authorized members of the research team.

### Statistical analysis

Proportions of patients with a given characteristic and means for quantitative data were computed with a 95% confidence interval unless otherwise stated. To identify variables associated with sensory extinction, a multivariable logistic regression analysis was performed. Sensory extinction was considered as the dependant variable. The independent variables were age (<60 years as reference), sex (female as reference), side of lesion (right versus left or bilateral), type of stroke (haemorrhagic versus ischaemic or mixed), stroke severity (NIHSS score <5 as reference), lesion volume (<2 mL as reference), time to first examination (>3 days as reference), and the presence of PVN. The choice of the dichotomization threshold for continuous variables was guided by their performance for the diagnosis of sensory extinction (see online resource 1). *p* values <0.05 were considered as significant. Statistical analysis was performed with the software STATA 13 (StataCorp LP, USA).

## Results

### Patients’ clinical characteristics

A total of 73 patients were recruited (38.4% women). Mean age was 62.3 years (95% CI 58.8–65.7) and mean NIHSS score was 1.6 (range 0–10, 95% CI 1.2–2.1). The mean time to first examination was 4.1 days (95% CI 3.5–4.8) and 78.1% (57/73) of the patients were examined within 5 days post-stroke. The prevalence of PVN was 23.3% (17/73; 95% CI 14.2–34.6). Patients’ baseline characteristics and performance on the neuropsychological tests are summarized in Tables 1 and 2, respectively.

**Table 1:** Baseline clinical characteristics of patients included

Factors studied	Sensory extinction		Total
	Yes^a^	No	
Sex			
Male	5 (11.1)	40	45
Female	5 (17.9)	23	28
Age	66.8 (56.6–77.0)	61.5 (57.7–65.3)	62.3 (58.8–65.7)
Side of lesion			
Right	3 (8.3)	33	36
Left	7 (21.2)	26	33
Bilateral lesions	0 (0)	4	4
Type of lesion			
Ischemic	9 (14.1)	55	64
Haemorrhagic	1 (11.1)	8	9
Stroke severity (NIHSS score)^b^	2.7 (0.8–4.6)	1.4 (1.0–1.8)	1.6 (1.2–2.1)
Lesion volume (mL)	17.3 (0.2–34.4)	6.5 (3.9–9.0)	8.0 (4.9–11.0)
Time to first examination	4.2 (3.5–4.9)	4.0 (3.2–4.8)	4.1 (3.5–4.8)
Handedness			
Right-handed	10 (15.2)	56	66
Left-handed	0 (0)	7	7
Visuospatial neglect			
No	6 (11.1)	50	56
Yes	4 (30.0)	13	17

^a^For categorical variables, the frequency is given with the percentage of total in the corresponding row. For continuous variables, the mean is given with the 95% confidence interval

^b^The NIHSS score ranged from 0 to 8 in patients with sensory extinction and from 0 to 10 in patients without sensory extinction

**Table 2:** Summarized results of the neuropsychological tests at baseline

Behavioural disorder and evaluation measures	Sensory extinction	
	Yes	No
Peripersonal visuospatial neglect^a^		
Ota’s gap detection task		
Number of targets omitted on the left	1.5 ± 1.3	0.1 ± 0.03
Number of targets omitted on the right	0.4 ± 0.4	0.1 ± 0.05
Total number of targets omitted	1.9 ± 1.7	0.2 ± 0.06
Line bisection task		
Rightward deviation in mm (5 cm line)	0.9 ± 0.3	0.9 ± 0.1
Leftward deviation in mm (5 cm line)	1.3 ± 0.7	1.4 ± 0.3
Rightward deviation in mm (20 cm line)	2.6 ± 0.9	3.4 ± 0.5
Leftward deviation in mm (20 cm line)	7.9 ± 2.4	2.7 ± 0.4
Sensory extinction^b^		
Homologous tactile extinction		
Unilateral stimulation on the left		
Without standardization	100.0 ± 0.0	100 ± 0.0
With standardization	96.0 ± 4.0	100 ± 0.0
Unilateral stimulation on the right		
Without standardization	100.0 ± 0.0	99.7 ± 0.3
With standardization	98.0 ± 2.0	99.7 ± 0.3
Bilateral stimulations		
Without standardization	98.0 ± 2.0	99.8 ± 0.2
With standardization	99 ± 1.0	100 ± 0.0
Heterologous tactile extinction		
Unilateral stimulation on the left		
Without standardization	100.0 ± 0.0	99.7 ± 0.3
With standardization	90.0 ± 10.0	100 ± 0.0
Unilateral stimulation on the right		
Without standardization	100.0 ± 0.0	99.4 ± 0.4
With standardization	90.0 ± 10.0	99.7 ± 0.3
Bilateral stimulations		
Without standardization	60.0 ± 13.0	98.4 ± 0.6
With standardization	69.0 ± 13.4	96.8 ± 0.8
Visual extinction		
Unilateral stimulation on the left		
Without standardization	100.0 ± 0.0	100.0 ± 0.0
Unilateral stimulation on the right		
Without standardization	100.0 ± 0.0	99.7 ± 0.3
Bilateral stimulations		
Without standardization	94.0 ± 4.0	99.7 ± 0.2
Auditory extinction		
Unilateral stimulation on the left		
Without standardization	100.0 ± 0.0	99.7 ± 0.3
With standardization	100.0 ± 0.0	100.0 ± 0.0
Unilateral stimulation on the right		
Without standardization	98.0 ± 2.0	99.7 ± 0.3
With standardization	100.0 ± 0.0	100.0 ± 0.0
Bilateral stimulations		
Without standardization	84.0 ± 8.1	97.5 ± 0.6
With standardization	99 ± 1.0	97.9 ± 1.1
Contralesional auditory-tactile cross-modal extinction		
Without standardization	74.0 ± 10.6	97.3 ± 0.7
With standardization	94.0 ± 5.0	99.0 ± 0.6
Ipsilesional auditory-tactile cross-modal extinction		
Without standardization	91.0 ± 5.5	99.2 ± 0.4
With standardization	97.0 ± 2.1	99.4 ± 0.3

^a^For peripersonal visuospatial neglect, the mean number of targets omitted or the mean deviation is presented with the standard error

^b^For sensory extinction, the mean percentage of correct detection for each type of stimulation is presented with the standard error

### Prevalence, risk factors, and time course of sensory extinction

**Fig. 1:**
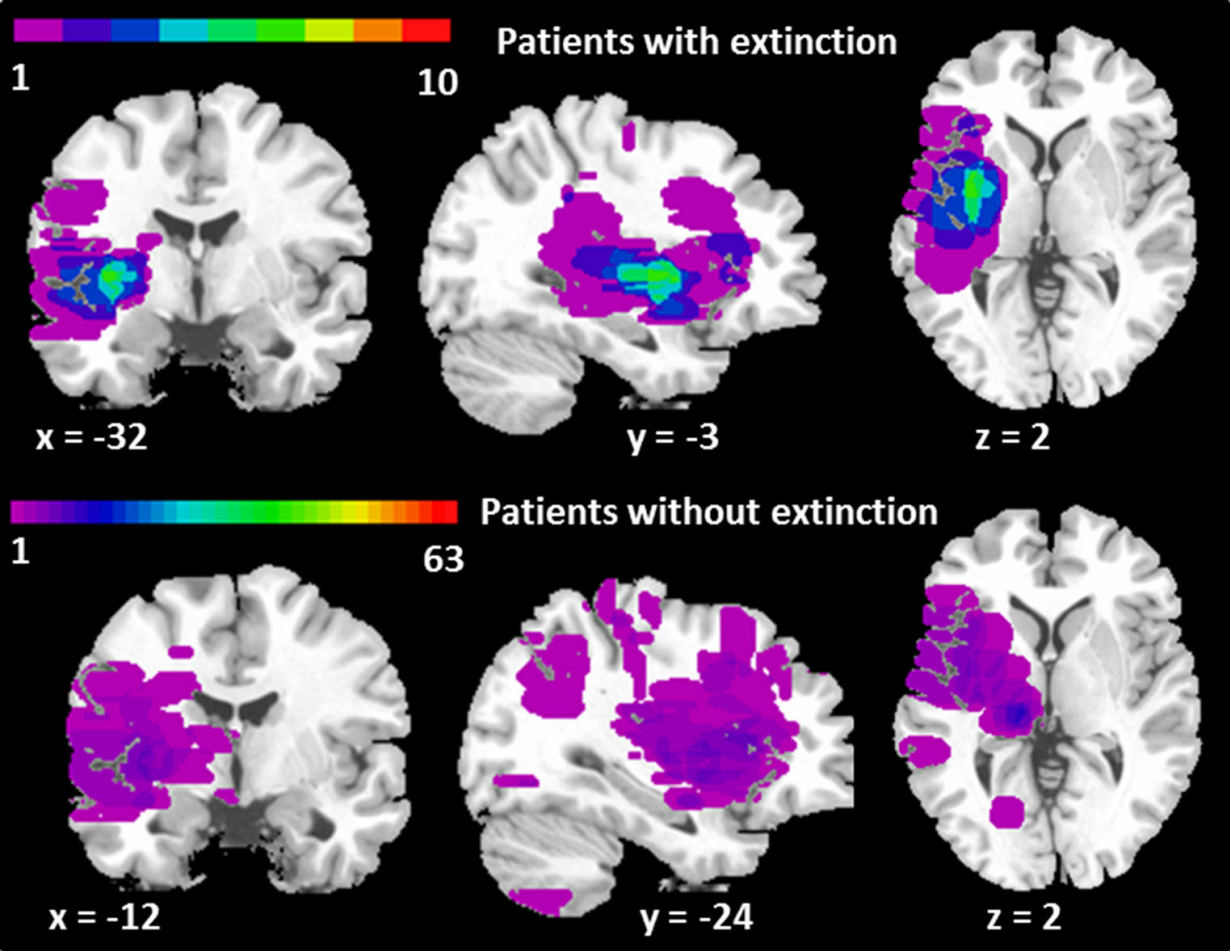
Lesion-overlap map for patients with and without sensory extinction. Lesions on the right side have been flipped to the left side to facilitate the global analysis. The coordinates (*x*, *y,* and *z*) of the region of maximum overlap are given in the Talairach’s 3D space. *Colour* codes represent the number of patients with damage to a given area, ranging from *purple* for areas affected in one patient only, to *red* for areas affected in all patients. In patients with sensory extinction, the region of maximum overlap (*green*) is affected in six patients (out of ten) and corresponds to the insular, putaminal, and the pallidal functional regions that had the highest region-involvement indices (Table 5)

Ten patients had at least one subtype of sensory extinction yielding an overall prevalence of 13.7% (6.8–23.8). The prevalence of each subtype of sensory extinction is given in Table 3. In the multivariable logistic regression analysis, a lesion volume ≥2 mL (adjusted OR = 38.88, *p* = 0.04) and presence of PVN (adjusted OR = 24.27, *p* = 0.04) were independent predictors of sensory extinction (Table 4). The insula, the putamen, and the pallidum were the brain regions most frequently involved in patients with sensory extinction, as shown in Table 5, and also in the lesion-overlap map (Fig. 1).

**Table 3:** Prevalence of various subtypes of sensory extinction

Extinction type	Presence of visuospatial neglect	Count	Total *n* (%, CI)^a^
Tactile			
Homologous	–	0	6 (8.2%, 3.1–17.0)
Heterologous	Yes	2	
	No	4^b^	
Auditory	No	3	3 (4.1%, 0.9–11.5)
Visual	Yes	1	1 (1.4%, 0–7.4)
Cross-modal (auditory-tactile)			
Ipsilesional	No	1	4 (5.5%, 1.5–13.4)
Contralesional	No	2	
Bilateral	Yes	1^c^	

^a^Estimated prevalence and confidence interval

^b^Among patients with heterologous tactile extinction, one also had auditory extinction and two had auditory-tactile cross-modal extinction

^c^The patient with bilateral auditory-tactile cross-modal extinction also had visual extinction

**Table 4:** Univariable and multivariable analyses of factors associated with sensory extinction

Characteristics^a^	Univariable model			Multivariable model		
	Crude OR	95% CI	*p*	Adjusted OR	95% CI	*p*
Male	0.58	0.15–2.20	0.42	1.77	0.27–11.41	0.55
Age ≥60 years	3.2	0.63–16.29	0.16	8.33	0.68–101.30	0.10
Right hemisphere lesion	0.39	0.09–1.64	0.20	0.27	0.04–2.04	0.21
Haemorrhagic stroke	0.76	0.09–6.86	0.81	0.46	0.03–7.56	0.60
NIHSS score ≥5	13.07	1.85–92.12	0.01	17.51	0.67–458.84	0.08
Lesion volume ≥2 mL	2.46	0.48–12.57	0.28	38.88	1.21–1245.17	0.04
Time to first examination ≤3 days	0.83	0.21–3.24	0.79	0.62	0.10–3.65	0.60
Visuospatial neglect	2.56	0.63–10.45	0.19	24.27	1.13–519.93	0.04
				Area under the ROC curve = 0.90 *R* ^2^ = 0.864		

^a^The handedness was not included in the model, because all the patients with sensory extinction are right-handed

**Table 5:** Overview of the functional brain regions damaged in patients with at least one subtype of sensory extinction (*n* = 10)

Side of lesion^a^	Fr	Ins	Rol	Par	Temp	Occ	Thal	CN	Put	Pal	IC	BS	C
RSS (left)	1	4	2	3	3	0	0	1	3	4	3	1	0
RSS (right)	1	1	2	0	1	0	0	0	2	1	0	0	0
Global RSS	2	5	4	3	4	0	0	1	5	5	3	1	0
Global RII	0.06	0.15	0.12	0.09	0.12	0.00	0.00	0.03	0.15	0.15	0.09	0.03	0.00

*H* handedness (*R* right-handed, *L* left-handed), *Fr* frontal, *Ins* insular, *Rol* rolandic, *Par* parietal, *Temp* temporal, *Occ* occipital, *Thal* thalamus, *CN* caudate nucleus, *Put* putamen, *Pall* pallidum, *IC* internal capsule, *BS* brainstem, *C* cerebellum, *RSS* region-specific score, *RII* region-involvement index

^a^There were seven patients with left hemisphere lesion and three patients with right hemisphere lesion

Sensory extinction was found in three patients at visits 2 and 3. Two of these patients had heterologous tactile extinction and a left hemisphere lesion, while the third had a right hemisphere lesion with visual extinction and ipsilesional auditory-tactile cross-modal extinction. No case of sensory extinction was found at visit 4 or beyond, which is 15 days after the first examination. The results of the neuropsychological evaluation during follow-up visits are summarized in Table 6.

**Table 6:** Neuropsychological outcome of patients with sensory extinction at visit 1

Patient ID	Side of lesion	Stroke severity (NIHSS score)	Lesion volume (mL)	Visit 1		Visit 2		Visit 3		Visit 4	
				Extinction	PVN	Extinction	PVN	Extinction	PVN	Extinction	PVN
18	Right	0	2.4	Yes	No	No	No	No	No	No	No
37^a^	Right	1	68.8	Yes	Yes	Yes	Yes	Yes	Yes	No	No
43	Right	2	2.1	Yes	No	No	No	No	No	No	No
101	Left	6	1.9	Yes	Yes	No	Yes	No	No	No	No
30^a^	Left	8	50.7	Yes	No	Yes	No	Yes	No	No	No
50	Left	2	1.2	Yes	Yes	No	No	No	No	No	No
22^a^	Left	1	5.1	Yes	No	Yes	No	Yes	No	No	No
6	Left	1	24.3	Yes	No	No	No	No	No	No	No
87	Left	5	3.7	Yes	Yes	No	Yes	No	Yes	No	No
25	Left	1	12.9	Yes	No	No	No	No	No	No	No

^a^Patients with sensory extinction at visits 2 and 3

### Results with standardization

When using standardized testing procedures, the prevalence of sensory extinction was 8.2% (95% CI 3.08–17.04). There was 89% agreement between non-standardized and standardized tests regarding the diagnosis of sensory extinction (*κ* = 0.44). At baseline, there was no case of homologous tactile extinction, three cases of heterologous tactile extinction, one case of auditory extinction, two cases of contralesional auditory-tactile cross-modal extinction, and no case of ipsilesional auditory-tactile cross-modal extinction. The agreement between non-standardized and standardized procedures was 100% for homologous tactile extinction (*κ* = 1), 96% for heterologous tactile extinction (*κ* = 0.64), 96% for contralesional auditory-tactile cross-modal extinction (*κ* = 0.40), 97.3% for ipsilesional auditory-tactile cross-modal extinction (*κ* = 0), and 94.5% for auditory extinction (*κ* = 0).

## Discussion

This study was carried out to determine the prevalence, potential risk factors, and the time course of sensory extinction in acute stroke. Among the 73 patients included, 13.7% had at least one subtype of sensory extinction and all recovered completely within 15 days after the first examination. Lesion volume ≥2 mL and presence of PVN were independent predictors of sensory extinction. Given that the standardization of testing procedures did not significantly increase the diagnostic yield, the discussion will be based solely on results obtained with non-standardized procedures that are more representative of the real-life practice in the acute stroke setting.

Studies of sensory extinction and neglect in acute stroke are needed to clarify their pathophysiological relation. The major logistic challenge of our exploratory cohort study was to develop a simple and practical, yet scientifically valid evaluation of sensory extinction in the acute stroke setting where disorders of attention and language are highly prevalent. Being aware of the fact that the pathophysiology of sensory extinction may involve directional and non-directional deficits of attention (de Haan et al. 2015), the critical issue was rather to minimize the false positive rate of extinction than to completely eliminate any single attentional deficit in patients before carrying out the neuropsychological tests. We believe that the following measures helped us to reasonably achieve this goal, though at the cost of lower inclusion rates in the group of moderate and severe strokes: (1) stringent selection criteria to exclude all patients in whom reduced alertness would have been a major confounder (elderly patients with pre-existing cognitive decline, patients with NIHSS >20 on the examination day), (2) a small number of trained and assessed examiners to ensure consistency of results, and (3) standardized sets of stimuli with cutoffs to eliminate false positives due to either random fluctuations of patients’ alertness during the assessment, random asymmetry of the strength of stimuli or random temporal asynchrony of stimulus onset, and termination during bilateral stimulations.

In this study, patients with bigger lesions were more likely to have at least one subtype of sensory extinction. This result may be explained by the fact that bigger lesions affect several functional brain regions in the human attentional network thus having a greater impact on the attentional capacity. As a consequence, the predominance of patients with small lesions in our sample suggests that the real prevalence of sensory extinction is underestimated. The independent association between sensory extinction and visuospatial neglect found in this work could be considered as an additional argument to support the hypothesis that there is some overlap in their pathophysiology. Further studies in the acute stroke setting are expected to disentangle the complex relation between these deficits. Unexpectedly, the side of the brain lesion was not significantly associated with the presence of sensory extinction. The previous studies of visual extinction have reported a higher prevalence in patients with right brain lesions, as is the case for visuospatial neglect (Becker and Karnath 2007; de Haan et al. 2012). There are three hypotheses that could explain this discrepancy.

First, it is possible that some patients with right hemisphere lesions (especially those involving the parietal lobe) and severe sensory neglect were mistakenly classified as having hemihypoesthesia or hemianesthesia upon admission and, therefore, not included in our study. These patients could, therefore, not undergo further testing for tactile, visual, or auditory extinction. Together with the stringent selection criteria, the exclusion of some patients with right hemisphere lesions could account for the low frequency of visual extinction leading to an underestimation of the overall prevalence of sensory extinction in our sample. Indeed, it is known that visual extinction, like neglect, is more common in patients with right hemisphere lesions—explaining high rates usually reported in studies focusing on patients with right brain lesions (Umarova et al. 2011; Vallar et al. 1994; Vossel et al. 2011). This is thought to result from the specialization of right hemisphere, and, specifically, the right parietal lobe, for visuospatial processing (Kinsbourne 1977; Mesulam 1981; Weintraub and Mesulam 1987).

Second, the lack of association between lesion side and sensory extinction could possibly be explained by a high prevalence of lacunar infarcts and lesions of the anterior circulation in our sample. In fact, it has been previously reported that the predominance of right hemisphere lesions in patients with sensory extinction is only observed for strokes affecting the middle cerebral artery and posterior cerebral artery territories and not for strokes affecting other vascular territories (Chechlacz et al. 2014). A predominance of small deep brain lesions in our sample (Table 5) would be coherent with the low mean NIHSS score and would again be a consequence of both the stringent selection process and the complex testing procedures used.

Third, the statistical analyses performed here include all types of sensory extinction, while the relation between the side of the brain lesion and sensory extinction could vary depending on the subtype of extinction considered. Likewise, the relation between neglect and sensory extinction might not be the same depending on the modality of neglect and the subtype of sensory extinction considered. This highlights the necessity to adapt selection criteria to the specific association under investigation. Further studies with larger sample size are warranted to allow for more subtle subgroup analyses before definitive conclusions could be made.

All patients with extinctions at visit 1 recovered within 15 days after the first examination. This rapid recovery might also be explained by the predominance of small subcortical lesions in our sample. Several mechanisms could be involved in this rapid recovery: (1) restoration of perfusion to penumbral regions rendered temporarily non-functional but not permanently injured by moderate degrees of ischemia, (2) resolution of cytotoxic oedema responsible for compression of tissues surrounding the infarct, (3) unmasking of redundant underused neural pathways, and (4) the early neural repair and network reorganization (Dobkin 1996).

This study has several strengths: the early recruitment of patients with acute stroke, use of repeated testing that increases the reliability of results, assessment of multiple subtypes of sensory extinction that increases the sensitivity of the screening, and longitudinal follow-up of patients with sensory extinction that allowed us to report the duration of this symptom for the first time. The lack of a voxel-based lesion statistical mapping (VLSM) analysis (Saj et al. 2012) is a major limitation of this study. Such analysis would have helped to refine our understanding of the relation between sensory extinction and the location of acute brain lesions. However, the validity of a post hoc analysis of the neuroanatomical correlates of sensory extinction would have been questionable given the low prevalence of sensory extinction in our sample. Moreover, such analysis would rely on the assumption that all subtypes of sensory extinction have the same anatomical substrate which would be highly speculative. A second limitation is the absence of a measure of interrater agreement for the neuropsychological tests. However, given that this study was carried out in the acute stroke setting with patients under physical and emotional stresses due to the diagnosis, the treatment, and the multiple paraclinical examinations and clinical trials going on at the same time, it was neither practically feasible nor ethically acceptable to have the same examinations performed by all three examiners at the same time for each visit. A third limitation is the lack of information on the neuropsychological rehabilitation programmes that could have influenced the time course of extinction in our cohort. Nevertheless, given that most of our patients had a mild stroke, it is unlikely that they have received specific rehabilitation therapies that could significantly interfere with our results. Indeed, all patients benefited for the standard stroke management protocol at the Geneva stroke unit. In this protocol, a specific neuropsychological rehabilitation programme is implemented only if it is deemed indispensable for the recovery. Other limitations are the small sample size and the heterogeneity in the time to the first examination. Nearly, 22% of our patients have had their first examination beyond the fifth day after stroke onset and it is not possible to know if they had sensory extinction in the early days that had been missed leading to underestimation of the overall prevalence. The delay is explained by various factors related to the acute stroke setting: time to obtain the informed consent, availability of the patients and the examiners, interference with the clinical management of the patient, and temporary stay in the intensive care unit before transfer into the stroke unit.

In conclusion, our study shows that sensory extinction is a rare and transient phenomenon in patients with mild acute stroke. Our results also indicate that the presence of PVN and lesion volume greater than 2 mL are independent predictors of sensory extinction. Accurately, determining the prevalence of sensory extinction in the acute stroke setting is difficult because of the concomitant presence of disorders of alertness or non-spatial attention and the low inclusion rates due to clinical status and higher tendency to withhold consent. As these results were obtained in a small sample with most patients having a low NIHSS score, they need to be confirmed in a larger cohort using more inclusive selection criteria adapted to the clinical context and the specific subtype of extinction investigated to avoid selection bias. The following questions could also be addressed in upcoming studies: (1) Do all subtypes of extinction have the same neural correlates? (2) Does extinction appear in patients with severe visuospatial neglect as they recover, suggesting that there is an overlap in their pathophysiology?

## Electronic supplementary material

Below is the link to the electronic supplementary material.

**Online Resource 1** Performance of various clinical characteristics for the diagnosis of sensory extinction: stroke severity, lesion volume, time to first examination, and age. The point of the ROC curve maximizing the sensitivity and the specificity was chosen as the dichotomization threshold for the continuous variable considered. For the time to first examination, we used 3 days rather than 4, because 24-, 48- and 72-h time windows are most commonly used in the routine clinical practice. (DOCX 44 kb)
