# Supplementary material for: An exploratory cohort study of sensory extinction in acute stroke: prevalence, risk factors, and time course
Source: J Neural Transm (Vienna). 2016 Dec 9;124(4):483–94. doi: 10.1007/s00702-016-1663-x (PMC5357267; doi:10.1007/s00702-016-1663-x)
Supplement: Supplementary file 1 — Online Resource 1 Performance of various clinical characteristics for the diagnosis of sensory extinction: stroke severity, lesion volume, time to first examination, and age. The point of the ROC curve maximizing the sensitivity and the specificity was chosen as the dichotomization threshold for the continuous variable considered. For the time to first examination, we used 3 days rather than 4, because 24-, 48- and 72-h time windows are most commonly used in the routine clinical practice. (DOCX 44 kb) [file 702_2016_1663_MOESM1_ESM.docx]

**PERFORMANCE OF VARIOUS CLINICAL CHARACTERISTICS FOR THE DIAGNOSIS OF SENSORY EXTINCTION**

**1. Age (years)**

| Cutpoint | Sensitivity | Specificity | Correctly Classified | LR+ | LR- |
| --- | --- | --- | --- | --- | --- |
| ( >= 29 ) | 100.00% | 0.00% | 13.70% | 1.0000 |  |
| ( >= 36 ) | 100.00% | 1.59% | 15.07% | 1.0161 | 0.0000 |
| ( >= 37 ) | 100.00% | 4.76% | 17.81% | 1.0500 | 0.0000 |
| ( >= 41 ) | 100.00% | 7.94% | 20.55% | 1.0862 | 0.0000 |
| ( >= 43 ) | 100.00% | 9.52% | 21.92% | 1.1053 | 0.0000 |
| ( >= 44 ) | 100.00% | 11.11% | 23.29% | 1.1250 | 0.0000 |
| ( >= 45 ) | 80.00% | 15.87% | 24.66% | 0.9509 | 1.2600 |
| ( >= 46 ) | 80.00% | 17.46% | 26.03% | 0.9692 | 1.1455 |
| ( >= 47 ) | 80.00% | 19.05% | 27.40% | 0.9882 | 1.0500 |
| ( >= 48 ) | 80.00% | 23.81% | 31.51% | 1.0500 | 0.8400 |
| ( >= 49 ) | 80.00% | 25.40% | 32.88% | 1.0723 | 0.7875 |
| ( >= 50 ) | 80.00% | 28.57% | 35.62% | 1.1200 | 0.7000 |
| ( >= 54 ) | 80.00% | 31.75% | 38.36% | 1.1721 | 0.6300 |
| ( >= 55 ) | 80.00% | 33.33% | 39.73% | 1.2000 | 0.6000 |
| ( >= 56 ) | 80.00% | 34.92% | 41.10% | 1.2293 | 0.5727 |
| ( >= 57 ) | 80.00% | 39.68% | 45.21% | 1.3263 | 0.5040 |
| ( >= 59 ) | 80.00% | 41.27% | 46.58% | 1.3622 | 0.4846 |
| ( >= 61 ) | 80.00% | 44.44% | 49.32% | 1.4400 | 0.4500 |
| ( >= 62 ) | 80.00% | 46.03% | 50.68% | 1.4824 | 0.4345 |
| ( >= 63 ) | 70.00% | 50.79% | 53.42% | 1.4226 | 0.5906 |
| ( >= 64 ) | 60.00% | 50.79% | 52.05% | 1.2194 | 0.7875 |
| ( >= 66 ) | 60.00% | 53.97% | 54.79% | 1.3034 | 0.7412 |
| ( >= 67 ) | 50.00% | 57.14% | 56.16% | 1.1667 | 0.8750 |
| ( >= 68 ) | 50.00% | 58.73% | 57.53% | 1.2115 | 0.8514 |
| ( >= 70 ) | 50.00% | 63.49% | 61.64% | 1.3696 | 0.7875 |
| ( >= 72 ) | 40.00% | 65.08% | 61.64% | 1.1455 | 0.9220 |
| ( >= 73 ) | 40.00% | 69.84% | 65.75% | 1.3263 | 0.8591 |
| ( >= 74 ) | 40.00% | 71.43% | 67.12% | 1.4000 | 0.8400 |
| ( >= 75 ) | 30.00% | 73.02% | 67.12% | 1.1118 | 0.9587 |
| ( >= 76 ) | 30.00% | 77.78% | 71.23% | 1.3500 | 0.9000 |
| ( >= 77 ) | 30.00% | 84.13% | 76.71% | 1.8900 | 0.8321 |
| ( >= 78 ) | 30.00% | 85.71% | 78.08% | 2.1000 | 0.8167 |
| ( >= 79 ) | 20.00% | 88.89% | 79.45% | 1.8000 | 0.9000 |
| ( >= 82 ) | 20.00% | 92.06% | 82.19% | 2.5200 | 0.8690 |
| ( >= 83 ) | 10.00% | 92.06% | 80.82% | 1.2600 | 0.9776 |
| ( >= 85 ) | 10.00% | 93.65% | 82.19% | 1.5750 | 0.9610 |
| ( >= 86 ) | 0.00% | 96.83% | 83.56% | 0.0000 | 1.0328 |
| ( >= 87 ) | 0.00% | 98.41% | 84.93% | 0.0000 | 1.0161 |
| ( > 87 ) | 0.00% | 100.00% | 86.30% |  | 1.0000 |

**2. Stroke severity (NIHSS score)**

| Cutpoint | Sensitivity | Specificity | Correctly Classified | LR+ | LR- |
| --- | --- | --- | --- | --- | --- |
| ( >= 0 ) | 100.00% | 0.00% | 13.70% | 1.0000 |  |
| ( >= 1 ) | 90.00% | 36.51% | 43.84% | 1.4175 | 0.2739 |
| ( >= 2 ) | 50.00% | 58.73% | 57.53% | 1.2115 | 0.8514 |
| ( >= 3 ) | 30.00% | 82.54% | 75.34% | 1.7182 | 0.8481 |
| ( >= 4 ) | 30.00% | 90.48% | 82.19% | 3.1500 | 0.7737 |
| ( >= 5 ) | 30.00% | 96.83% | 87.67% | 9.4500 | 0.7230 |
| ( >= 6 ) | 20.00% | 96.83% | 86.30% | 6.3000 | 0.8262 |
| ( >= 8 ) | 10.00% | 98.41% | 86.30% | 6.3000 | 0.9145 |
| ( >= 10 ) | 0.00% | 98.41% | 84.93% | 0.0000 | 1.0161 |
| ( > 10 ) | 0.00% | 100.00% | 86.30% |  | 1.0000 |

**3. Lesion volume (mL)**

| Cutpoint | Sensitivity | Specificity | Correctly Classified | LR+ | LR- |
| --- | --- | --- | --- | --- | --- |
| ( >= .3 ) | 100.00% | 0.00% | 13.70% | 1.0000 |  |
| ( >= .6 ) | 100.00% | 1.59% | 15.07% | 1.0161 | 0.0000 |
| ( >= .8 ) | 100.00% | 3.17% | 16.44% | 1.0328 | 0.0000 |
| ( >= 1 ) | 100.00% | 6.35% | 19.18% | 1.0678 | 0.0000 |
| ( >= 1.1 ) | 100.00% | 7.94% | 20.55% | 1.0862 | 0.0000 |
| ( >= 1.2 ) | 100.00% | 9.52% | 21.92% | 1.1053 | 0.0000 |
| ( >= 1.3 ) | 90.00% | 12.70% | 23.29% | 1.0309 | 0.7875 |
| ( >= 1.4 ) | 90.00% | 22.22% | 31.51% | 1.1571 | 0.4500 |
| ( >= 1.5 ) | 90.00% | 25.40% | 34.25% | 1.2064 | 0.3938 |
| ( >= 1.6 ) | 90.00% | 28.57% | 36.99% | 1.2600 | 0.3500 |
| ( >= 1.7 ) | 90.00% | 33.33% | 41.10% | 1.3500 | 0.3000 |
| ( >= 1.8 ) | 90.00% | 36.51% | 43.84% | 1.4175 | 0.2739 |
| ( >= 1.9 ) | 90.00% | 38.10% | 45.21% | 1.4538 | 0.2625 |
| ( >= 2 ) | 80.00% | 38.10% | 43.84% | 1.2923 | 0.5250 |
| ( >= 2.1 ) | 80.00% | 41.27% | 46.58% | 1.3622 | 0.4846 |
| ( >= 2.2 ) | 70.00% | 41.27% | 45.21% | 1.1919 | 0.7269 |
| ( >= 2.3 ) | 70.00% | 42.86% | 46.58% | 1.2250 | 0.7000 |
| ( >= 2.4 ) | 70.00% | 44.44% | 47.95% | 1.2600 | 0.6750 |
| ( >= 2.5 ) | 60.00% | 44.44% | 46.58% | 1.0800 | 0.9000 |
| ( >= 2.6 ) | 60.00% | 46.03% | 47.95% | 1.1118 | 0.8690 |
| ( >= 2.8 ) | 60.00% | 47.62% | 49.32% | 1.1455 | 0.8400 |
| ( >= 3.1 ) | 60.00% | 49.21% | 50.68% | 1.1812 | 0.8129 |
| ( >= 3.5 ) | 60.00% | 53.97% | 54.79% | 1.3034 | 0.7412 |
| ( >= 3.7 ) | 60.00% | 55.56% | 56.16% | 1.3500 | 0.7200 |
| ( >= 3.8 ) | 50.00% | 55.56% | 54.79% | 1.1250 | 0.9000 |
| ( >= 3.9 ) | 50.00% | 57.14% | 56.16% | 1.1667 | 0.8750 |
| ( >= 4.4 ) | 50.00% | 58.73% | 57.53% | 1.2115 | 0.8514 |
| ( >= 4.7 ) | 50.00% | 60.32% | 58.90% | 1.2600 | 0.8289 |
| ( >= 5.1 ) | 50.00% | 61.90% | 60.27% | 1.3125 | 0.8077 |
| ( >= 5.8 ) | 40.00% | 61.90% | 58.90% | 1.0500 | 0.9692 |
| ( >= 5.9 ) | 40.00% | 65.08% | 61.64% | 1.1455 | 0.9220 |
| ( >= 6 ) | 40.00% | 68.25% | 64.38% | 1.2600 | 0.8791 |
| ( >= 6.1 ) | 40.00% | 69.84% | 65.75% | 1.3263 | 0.8591 |
| ( >= 6.2 ) | 40.00% | 73.02% | 68.49% | 1.4824 | 0.8217 |
| ( >= 6.5 ) | 40.00% | 74.60% | 69.86% | 1.5750 | 0.8043 |
| ( >= 6.7 ) | 40.00% | 77.78% | 72.60% | 1.8000 | 0.7714 |
| ( >= 6.8 ) | 40.00% | 79.37% | 73.97% | 1.9385 | 0.7560 |
| ( >= 8 ) | 40.00% | 80.95% | 75.34% | 2.1000 | 0.7412 |
| ( >= 8.2 ) | 40.00% | 82.54% | 76.71% | 2.2909 | 0.7269 |
| ( >= 8.3 ) | 40.00% | 84.13% | 78.08% | 2.5200 | 0.7132 |
| ( >= 8.5 ) | 40.00% | 85.71% | 79.45% | 2.8000 | 0.7000 |
| ( >= 12.9 ) | 40.00% | 87.30% | 80.82% | 3.1500 | 0.6873 |
| ( >= 13.2 ) | 30.00% | 87.30% | 79.45% | 2.3625 | 0.8018 |
| ( >= 13.6 ) | 30.00% | 88.89% | 80.82% | 2.7000 | 0.7875 |
| ( >= 16.2 ) | 30.00% | 90.48% | 82.19% | 3.1500 | 0.7737 |
| ( >= 18.1 ) | 30.00% | 92.06% | 83.56% | 3.7800 | 0.7603 |
| ( >= 24.3 ) | 30.00% | 93.65% | 84.93% | 4.7250 | 0.7475 |
| ( >= 32.1 ) | 20.00% | 93.65% | 83.56% | 3.1500 | 0.8542 |
| ( >= 33.6 ) | 20.00% | 95.24% | 84.93% | 4.2000 | 0.8400 |
| ( >= 38.2 ) | 20.00% | 96.83% | 86.30% | 6.3000 | 0.8262 |
| ( >= 50.7 ) | 20.00% | 98.41% | 87.67% | 12.6000 | 0.8129 |
| ( >= 59.4 ) | 10.00% | 98.41% | 86.30% | 6.3000 | 0.9145 |
| ( >= 68.8 ) | 10.00% | 100.00% | 87.67% |  | 0.9000 |
| ( > 68.8 ) | 0.00% | 100.00% | 86.30% |  | 1.0000 |

**4. Time to first examination (days)**

| Cutpoint | Sensitivity | Specificity | Correctly Classified | LR+ | LR- |
| --- | --- | --- | --- | --- | --- |
|  |  |  |  |  |  |
| ( >= 0 ) | 100.00% | 0.00% | 13.70% | 1.0000 |  |
| ( >= 1 ) | 100.00% | 1.59% | 15.07% | 1.0161 | 0.0000 |
| ( >= 2 ) | 100.00% | 14.29% | 26.03% | 1.1667 | 0.0000 |
| ( >= 3 ) | 100.00% | 28.57% | 38.36% | 1.4000 | 0.0000 |
| ( >= 4 ) | 60.00% | 44.44% | 46.58% | 1.0800 | 0.9000 |
| ( >= 5 ) | 30.00% | 63.49% | 58.90% | 0.8217 | 1.1025 |
| ( >= 6 ) | 10.00% | 76.19% | 67.12% | 0.4200 | 1.1812 |
| ( >= 7 ) | 0.00% | 85.71% | 73.97% | 0.0000 | 1.1667 |
| ( >= 8 ) | 0.00% | 90.48% | 78.08% | 0.0000 | 1.1053 |
| ( >= 10 ) | 0.00% | 93.65% | 80.82% | 0.0000 | 1.0678 |
| ( >= 12 ) | 0.00% | 96.83% | 83.56% | 0.0000 | 1.0328 |
| ( >= 15 ) | 0.00% | 98.41% | 84.93% | 0.0000 | 1.0161 |
| ( > 15 ) | 0.00% | 100.00% | 86.30% |  | 1.0000 |
